# A citation-based, author- and age-normalized, logarithmic index for evaluation of individual researchers independently of publication counts

**DOI:** 10.12688/f1000research.7070.1

**Published:** 2015-09-22

**Authors:** Aleksey V. Belikov, Vitaly V. Belikov

**Affiliations:** 1 Otto-von-Guericke-Universität, Magdeburg, Germany; 2 Water Problems Institute, Russian Academy of Sciences, Moscow, Russian Federation

**Keywords:** Research, scientific, metrics, impact, weighted, bibliometric, scientometric, Publish or Perish

## Abstract

The use of citation metrics for evaluation of individual researchers has dramatically increased over the last decade. However, currently existing indices either are based on misleading premises or are cumbersome to implement. This leads to poor assessment of researchers and creates dangerous trends in science, such as overproduction of low quality articles. Here we propose an index (namely, the L-index) that does not depend on the number of publications, accounts for different co-author contributions and age of publications, and scales from 0.0 to 9.9. Moreover, it can be calculated with the help of freely available software.

## Introduction

There is an ever-present need to evaluate researchers’ performance, because resources are limited and contenders are numerous. Before the advent of journal impact factors (JIFs, ^1^), all evaluations were performed via peer review. Although JIFs were intended for librarians to decide which journals to subscribe to, they have become a commonly used proxy for the quality of journal articles ^2^. However, the distribution of citations to individual articles within a journal is highly skewed. Twenty five percent of the most highly cited articles can account for 90% of a journal’s IF ^3^. The rest of the articles receive a few citations each, if any. Thus, using JIFs for assessing the quality of individual articles and, further, for evaluating researchers is categorically not recommended ^4^.

The rapid development of the internet and electronic citation databases, such as PubMed, Google Scholar, Web of Science, Scopus, CiteSeer and others, has made it easier to count citations of individual articles. It is now possible to automatically calculate the total number of citations that the publications of a given researcher have accumulated. However, these numbers can range from 1 to 100,000 and, obviously, do not represent the equal variation in researchers’ capabilities. For example, human IQ scores vary only about 2–4 fold ^5^.

There are two main reasons why the total number of citations cannot be used to adequately compare individual researchers. First, there is usually more than one author for each publication. Some of the most highly cited articles, such as reports from experiments on particle accelerators ^6^ or from genome sequencings ^7^, and guidelines for medical practitioners ^8^, have from tens to hundreds of co-authors, usually listed in alphabetical order. Thus, it is inadequate to assign all the thousands of citations to each of those authors. A similar situation is when a researcher operates some very expensive and thus rare equipment, and is listed on papers of all other researchers who perform experiments on that equipment ^9^. It is clear that the researcher also does not deserve *all* citations of those papers, as his contribution is purely technical. Many ways to divide citations between co-authors have been proposed ^10, 11^, but the only practical way is to split them equally, i.e. to assign each co-author **1/ *n*** of the citations, where ***n*** is the total number of co-authors.

Another factor is that citations accumulate with time. A researcher who started his career 30 years ago will undoubtedly have more citations than a young postdoc, but this does not necessarily mean that the former is a better scientist. Moreover, a paper that has been highly cited is likely to be cited even more in the future ^12^. Thus, citations exhibit the behavior of preferential attachment, which results in their distribution according to the power law ^13^. These considerations make it necessary to adjust for the age of each publication, in order to properly assess *current* capabilities and impact of researchers, not their past successes, and to partially compensate for the preferential attachment. Dividing the number of citations by the age of the publication in years seems to be an adequate measure, as it mirrors the power law distribution that citations have.

Finally, a large variety of individual citation metrics have been proposed ^14^, the most widely disseminated of which is the h-index ^15^. The drawback of the majority of these metrics is that they take into consideration the number of publications. For example, the h-index can never exceed the total number of publications a scientist has. However, several researchers of undisputed scientific merit, such as Sir Isaac Newton, Gregor Mendel or Peter Higgs, have published only a few, however significant, works. This lack in the *number* of publications leads them to have h-indices of 4, 1 and 9, respectively, which are disparagingly low. All other derivatives of the h-index, as well as all indices that take into account publication counts, suffer from the same drawback and hence should never be used for evaluation purposes. However, they *are* used, promoting a grueling and futile quest for quantity of publications, at the expense of quality, reflected in the infamous “publish or perish” catchphrase ^16^. Fortunately, this issue has been recently called to public attention, most notably in the form of the San Francisco Declaration on Research Assessment, and some measures have been proposed ^17^.

Overall, there is an immense need for a simple but reliable indicator for individual researcher assessment. Here, we propose such an index, which accounts for different co-author contributions and age of publications, and does not depend on the number of publications. Moreover, it conveniently ranges from 0.0 to 9.9, and can be calculated with the help of freely available software.

## Methods

To address the concerns highlighted in the introduction of this article, we have set out to construct an index that accounts for different co-author contributions and age of publications. This has initially led us to the following formula:

$$I=\sum_{i=1}^{N}\frac{c_{i}}{a_{i}y_{i}}\;(1)$$

where *I* – *preliminary* index, *c _i_* – number of citations to *i*-th publication, *a _i_* – number of authors of *i*-th publication, *y _i_* – age in years of *i*-th publication, *N* – number of publications.

**Figure 1.**
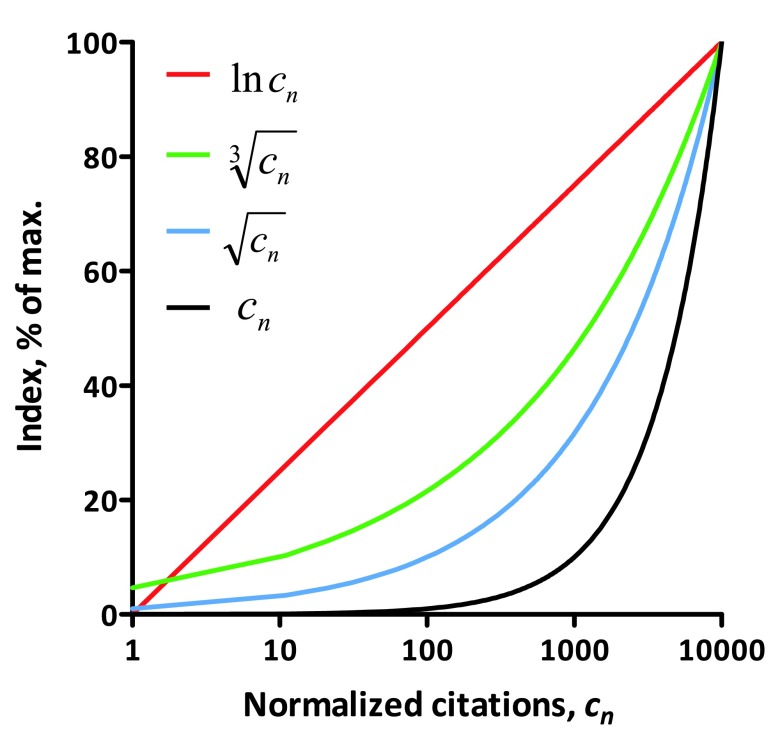
The natural logarithm function increases evenly across the citation range. The natural logarithm (ln ***c**_n_*), cube root $\left(\sqrt[{3}]{c_{n}}\right)$ and square root $\left(\sqrt{c_{n}}\right)$ functions of normalized citations ( ***c**_n_*) are shown.

We then decided to estimate the range of values that our preliminary index can have. First, we calculated *I* for a hypothetical PhD student who recently received the first citation to his first paper, with 5 authors: $I=\frac{1}{5\times 1}=0.2.$ Next, we calculated *I* for Albert Einstein and Charles Darwin, two of the most prominent and well-known scientists. To this aim, we utilized the freely available software *Publish or Perish*. This program imports citation data from *Google Scholar* and allows removal of irrelevant results, such as publications of homonym authors. A parameter *AWCRpA* (age-weighted citation rate per author) can be obtained from this program, and is equivalent to *I* from formula (1). The *AWCRpA* values for Einstein and Darwin, as calculated by *Publish or Perish* at the time of writing this article, were 6466 and 6178, respectively. Thus, even upon correcting for multiple authorship and age, citations vary by approximately 30,000 fold. As it is unlikely that the human brain can exhibit such a tremendous difference in its efficiency or talents, normalized citations should be mapped to a more appropriate scale, in order to make them more useful in meaningfully comparing researchers. It seems that a 1–10 scale is optimal, because it is closer to the true variation in human intellectual or other capabilities ^5^, and is widely used in various metrics, thus being more intuitive. The natural logarithm function appears to be ideal for this scaling purpose. Compared to the square root or the cube root, the natural logarithm allows better resolution of differences between the majority of scientists, with the exception of the most prominent ones ( Figure 1). To account for some of the negative values that arise when there is less than one normalized citation, the resulting index is increased by one point. In extreme cases where the index value still remains negative, it is advised to simply consider it as zero.

Finally, the formula for the **Logarithm index** (L-index) has become:

$$L=\ln\left(\sum_{i=1}^{N}\frac{c_{i}}{a_{i}y_{i}}\right)+1\;(2)$$

where *c _i_* – number of citations to *i*-th publication, *a _i_* – number of authors of *i*-th publication, *y _i_* – age in years of *i*-th publication, *N* – number of publications.

 When $\sum_{i=1}^{N}\frac{c_{i}}{a_{i}y_{i}}$ is calculated as *AWCRpA* in *Publish or Peris* *h*, formula (2) takes the form:

L=ln⁡AWCRpA+1(3)

Equation 3 has been used to obtain all L-index values in this article.

To calculate typical L-indices for a PhD student, a postdoc and a principal investigator (PI), we averaged the L-index values for 5 PhD students, 10 postdocs and 15 PIs that we personally know (see Supplementary Table 1).

## Results and discussion

**Figure 2.**
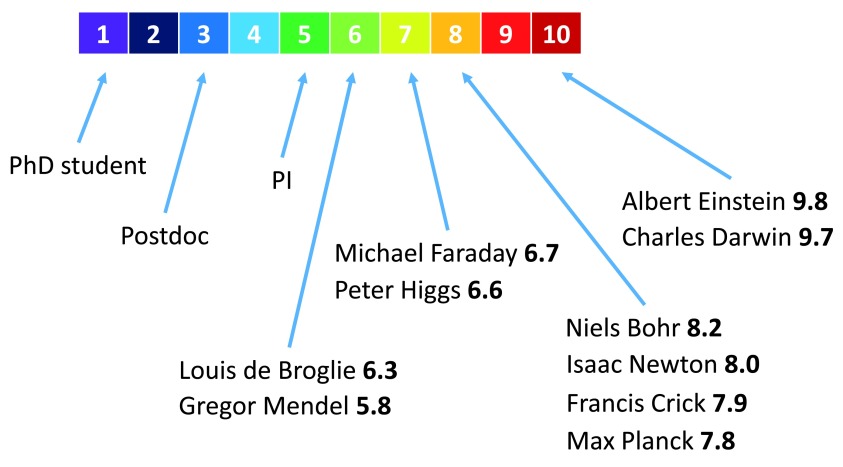
The L-index scale and notable examples. The typical range of the L-index is shown. The positions of 10 of the most famous scientists are indicated, along with their L-index scores. The positions of a typical PhD student, a postdoc and a principal investigator (PI) are also displayed. The raw data can be seen in Supplementary Table 1.

Figure 2 shows the typical scale of the L-index with the indication of the values for ten of some of the most prominent and widely recognized scientists, as well as the typical values for a PhD student, a postdoc and a principal investigator (PI) (see Supplementary Table 1).

It can be seen from this figure that the L-index adequately captures the intuitive ranking, i.e. PhD student < Postdoc < PI < … < Albert Einstein. Moreover, it allows the objective (or, at least, statistically averaged collective subjective) quantitative assessment of researchers, which is a virtue that traditional peer review cannot accomplish. However, in cases where L-indices of the applicants are equal up to one decimal place, we strongly suggest the use of peer review, involving thorough examination of their publications, rather than differentiation of scientists based on the second decimal place, to avoid false precision and statistical bias. In case of young researchers that have only a few citations, it is also advisable to use peer review, as the limited data do not allow for the statistically robust calculation of the citation index.

The L-index can increase or decrease with time, as it depends on the age of publications. Thus, it favors the impact of recent publications and gives a much needed advantage to younger researchers. However, if a scientist has made such a significant discovery that its impact only increases with time, his L-index will stay high regardless of the age of the publication. Perfect examples of this are Albert Einstein and Charles Darwin. Despite them ceasing to publish original work decades ago, their L-indices are still higher than those of the absolute majority of current researchers ( Figure 2).

The quantitative comparison of the L-index with other evaluation indices, such as the h-index, is purposefully avoided in this article, for the reason that those indices have been designed on different premises, such as to account for the number of publications. When evaluating the performance of a researcher, it should first be decided which parameter is considered adequate for the purpose – the number of publications, which does not tell anything about their quality, or the number of citations, which, however indirectly, indicates the impact that the publications have made. If the latter option is selected, the L-index can help to account for the effects of multiple co-authorship and aging of publications, and present the results in a simple and intuitive form.

## Supplementary material

**Table 1. The raw data that was used to calculate L-indices for Figure 2**.

*AWCRpA* were obtained from *Publish or Perish*, upon excluding publications of homonym authors. L-indices were calculated according to formula (3), using *Excel* formula editor and *LN* function. Average L-indices were calculated using *AVERAGE* function.
