# A citation-based, author- and age-normalized, logarithmic index for evaluation of individual researchers independently of publication counts

**DOI:** 10.12688/f1000research.7070.2

**Published:** 2025-06-02

**Authors:** Aleksey V. Belikov, Vitaly V. Belikov

**Affiliations:** 1 Otto-von-Guericke-Universität, Magdeburg, Germany; 2 Water Problems Institute, Russian Academy of Sciences, Moscow, Russian Federation

**Keywords:** Research, scientific, metrics, impact, weighted, bibliometric, scientometric, Publish or Perish

## Abstract

The use of citation metrics for evaluation of individual researchers has dramatically increased over the last decade. However, currently existing indices either are based on misleading premises or are cumbersome to implement. This leads to poor assessment of researchers and creates dangerous trends in science, such as overproduction of low quality articles. Here we propose an index (namely, the L-index) that does not depend on the number of publications, accounts for different co-author contributions and age of publications, and scales from 0.0 to 9.9. Moreover, it can be calculated with the help of freely available software.

## Introduction

There is an ever-present need to evaluate researchers’ performance, because resources are limited and contenders are numerous. Before the advent of journal impact factors (JIFs, ^1^), all evaluations were performed via peer review. Although JIFs were intended for librarians to decide which journals to subscribe to, they have become a commonly used proxy for the quality of journal articles ^2^. However, the distribution of citations to individual articles within a journal is highly skewed. Twenty five percent of the most highly cited articles can account for 90% of a journal’s IF ^3^. The rest of the articles receive a few citations each, if any. Thus, using JIFs for assessing the quality of individual articles and, further, for evaluating researchers is categorically not recommended ^4^.

The rapid development of the internet and electronic citation databases, such as PubMed, Google Scholar, Web of Science, Scopus, CiteSeer and others, has made it easier to count citations of individual articles. It is now possible to automatically calculate the total number of citations that the publications of a given researcher have accumulated. However, these numbers can range from 1 to 100,000 and, obviously, do not represent the equal variation in researchers’ capabilities. For example, human IQ scores vary only about 2–4 fold ^5^.

There are two main reasons why the total number of citations cannot be used to adequately compare individual researchers. First, there is usually more than one author for each publication. Some of the most highly cited articles, such as reports from experiments on particle accelerators ^6^ or from genome sequencings ^7^, and guidelines for medical practitioners ^8^, have from tens to hundreds of co-authors, usually listed in alphabetical order. Thus, it is inadequate to assign all the thousands of citations to each of those authors. A similar situation is when a researcher operates some very expensive and thus rare equipment, and is listed on papers of all other researchers who perform experiments on that equipment ^9^. It is clear that the researcher also does not deserve *all* citations of those papers, as his contribution is purely technical. Many ways to divide citations between co-authors have been proposed ^10, 11^, but the only practical way is to split them equally, i.e. to assign each co-author **1/ *n*** of the citations, where ***n*** is the total number of co-authors.

Another factor is that citations accumulate with time. A researcher who started his career 30 years ago will undoubtedly have more citations than a young postdoc, but this does not necessarily mean that the former is a better scientist. Moreover, a paper that has been highly cited is likely to be cited even more in the future ^12^. Thus, citations exhibit the behavior of preferential attachment, which results in their distribution according to the power law ^13^. These considerations make it necessary to adjust for the age of each publication, in order to properly assess *current* capabilities and impact of researchers, not their past successes, and to partially compensate for the preferential attachment. Dividing the number of citations by the age of the publication in years seems to be an adequate measure, as it mirrors the power law distribution that citations have.

Finally, a large variety of individual citation metrics have been proposed ^14^, the most widely disseminated of which is the h-index ^15^. The drawback of the majority of these metrics is that they take into consideration the number of publications. For example, the h-index can never exceed the total number of publications a scientist has. However, several researchers of undisputed scientific merit, such as Sir Isaac Newton, Gregor Mendel or Peter Higgs, have published only a few, however significant, works. This lack in the *number* of publications leads them to have h-indices of 4, 1 and 9, respectively, which are disparagingly low. All other derivatives of the h-index, as well as all indices that take into account publication counts, suffer from the same drawback and hence should never be used for evaluation purposes. However, they *are* used, promoting a grueling and futile quest for quantity of publications, at the expense of quality, reflected in the infamous “publish or perish” catchphrase ^16^. Fortunately, this issue has been recently called to public attention, most notably in the form of the San Francisco Declaration on Research Assessment, and some measures have been proposed ^17^.

Overall, there is an immense need for a simple but reliable indicator for individual researcher assessment. Here, we propose such an index, which accounts for different co-author contributions and age of publications, and does not depend on the number of publications. Moreover, it conveniently ranges from 0.0 to 9.9, and can be calculated with the help of freely available software.

## Methods

To address the concerns highlighted in the introduction of this article, we have set out to construct an index that accounts for different co-author contributions and age of publications. This has initially led us to the following formula:


$$I=\sum_{i=1}^{N}\frac{c_{i}}{a_{i}y_{i}}\;\text{(}1\text{)}$$

where *I* – *preliminary* index, *c _i_* – number of citations to *i*-th publication, *a _i_* – number of authors of *i*-th publication, *y _i_* – age in years of *i*-th publication, *N* – number of publications.

**Figure 1.**
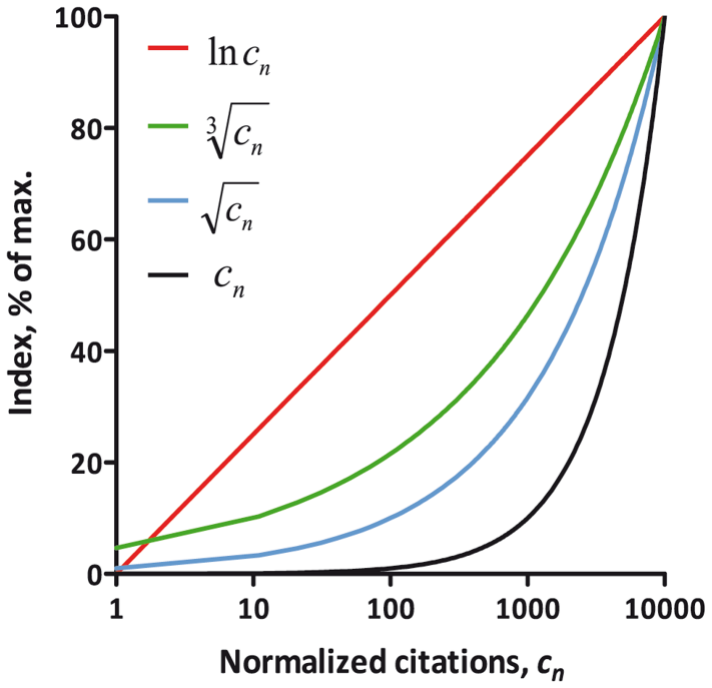
The natural logarithm function increases evenly across the citation range. The natural logarithm (ln ***c**_n_*), cube root $\left(\sqrt[{3}]{c_{n}}\right)$ and square root $\left(\sqrt{c_{n}}\right)$ functions of normalized citations ( ***c**_n_*) are shown.

We then decided to estimate the range of values that our preliminary index can have. First, we calculated *I* for a hypothetical PhD student who recently received the first citation to his first paper, with 5 authors: $I=\frac{1}{5\times 1}=0.2.$ Next, we calculated *I* for Geoffrey Hinton, a British-Canadian computer scientist, cognitive scientist, and Nobel laureate in physics, known for his work on artificial neural networks, with almost a million citations. To this aim, we utilized the freely available software *Publish or Perish*. This program imports citation data from *Google Scholar* author profiles. The parameter *AWCRpA* (age-weighted citation rate per author) can be obtained from this program, and is roughly equivalent to our preliminary index. The *AWCRpA* value for Hinton, as calculated by *Publish or Perish* on 8 ^th^ of May 2025, was 23875. Thus, even upon correcting for multiple authorship and age, the sum of citations varies by approximately 100000 fold. As it is unlikely that the human brain can exhibit such a tremendous difference in its efficiency or talents, the preliminary index should be mapped to a more appropriate scale, in order to make it more useful in meaningfully comparing researchers. It seems that a 1–10 scale is optimal, because it is closer to the true variation in human intellectual or other capabilities ^5^, and is widely used in various metrics, thus being more intuitive. The natural logarithm function appears to be ideal for this scaling purpose. Compared to the square root or the cube root, the natural logarithm allows better resolution of differences between the majority of scientists, with the exception of the most prominent ones ( Figure 1). To prevent the L-index from getting into the negative values when the preliminary index is less than one, the preliminary index is increased by one point before taking the logarithm.

Finally, the formula for the **Logarithm index** (L-index) became:


$$L=\text{ln}\left(\sum_{i=1}^{N}\frac{c_{i}}{a_{i}y_{i}}+1\right)\;\text{(}2\text{)}$$

where *c _i_* – number of citations to *i*-th publication, *a _i_* – number of authors of *i*-th publication, *y _i_* – age in years of *i*-th publication, *N* – number of publications.

 When $\sum_{i=1}^{N}\frac{c_{i}}{a_{i}y_{i}}$ is calculated as *AWCRpA* in *Publish or Peris* *h*, formula (2) takes the form:


L=ln(AWCRpA+1)(3)

*Publish or Perish*, while being an excellent piece of software, relies on scraping the Google Scholar profile publication lists, which display truncated lists of coauthors for many publications. This prevents calculation of the correct number of coauthors and hence the correct L-index. To allow precise L-index calculation, we created a Python script ^18^ that retrieves every publication from Google Scholar individually, each containing the full unabridged list of coauthors. It then counts the number of coauthors for each publication and calculates the L-index.

## Results and discussion

**Figure 2.**
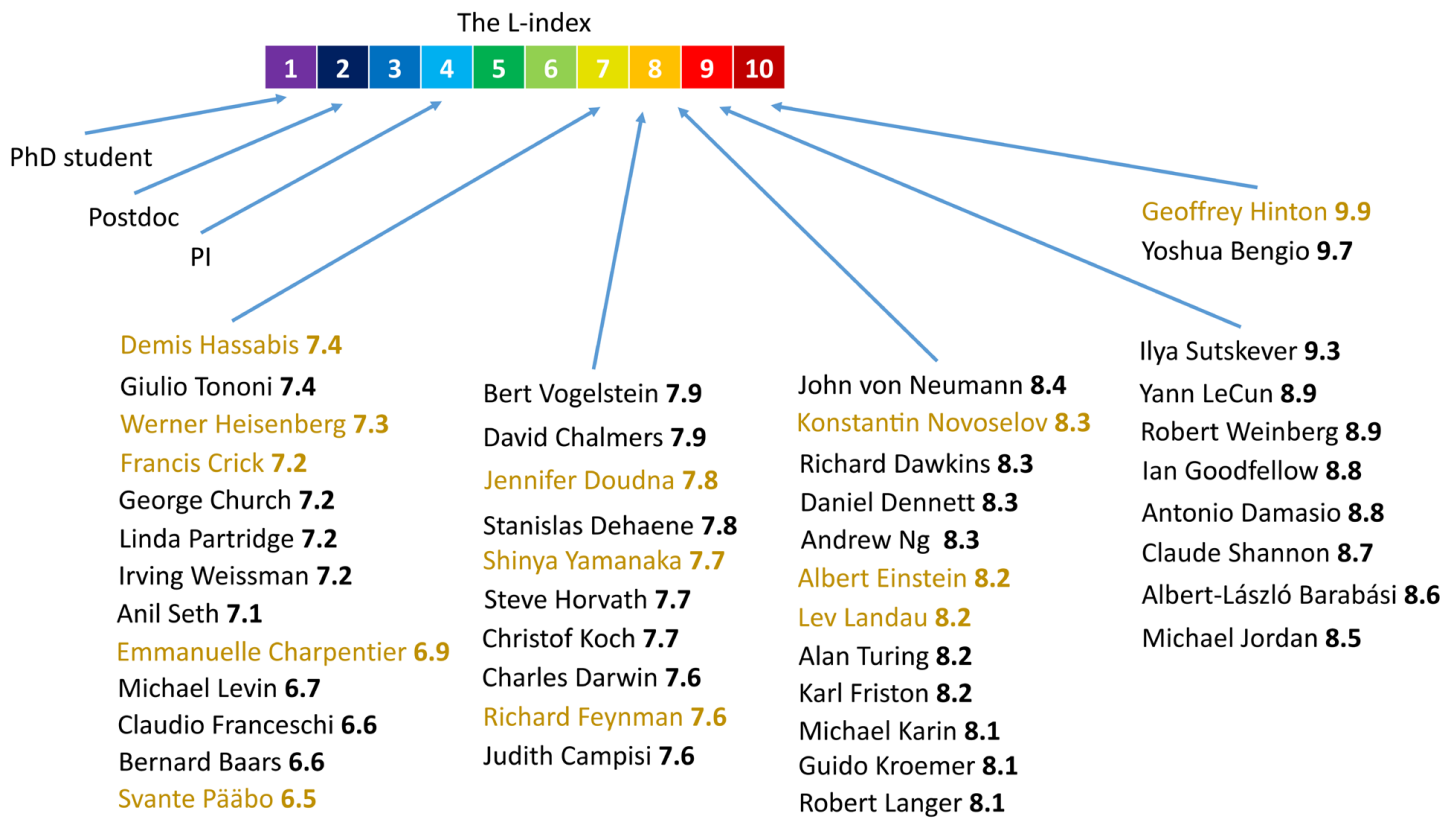
The L-index scale and notable examples. The typical range of the L-index is shown. L-index values of 45 prominent scientists in biomedicine, neuroscience, physics and computer science are shown. Nobel Prize laureates are indicated in gold. Approximate positions of a typical PhD student, postdoc and principal investigator (PI) are also displayed. The L-index values were calculated on the 8 ^th^ of May 2025 using the L-index Calculator ^18^ based on 100 most cited publications from publicly available Google Scholar profiles. The reports for each scientist containing top publications sorted by their contribution to the L-index are available as an external dataset ^19^.

Figure 2 shows the typical scale of the L-index with the values for 45 prominent scientists in biomedicine, neuroscience, physics and computer science, as well as the typical values for a PhD student, a postdoc and a principal investigator (PI). The L-index values were calculated on the 8 ^th^ of May 2025 using the L-index Calculator ^18^ based on 100 most cited publications from publicly available Google Scholar profiles. The reports for each scientist, containing top publications sorted by their contribution to the L-index, are available as an external dataset ^19^. To estimate typical L-indices for a PhD student, a postdoc and a principal investigator (PI), we averaged the L-index values for 5 PhD students, 10 postdocs and 15 PIs that we personally know. 

It can be seen from this figure that the L-index adequately captures the intuitive ranking, i.e. PhD student < Postdoc < PI < … < Nobel Laureates. Moreover, it allows an objective (or, at least, statistically averaged collective subjective) quantitative assessment of researchers, which is a virtue that traditional peer review cannot accomplish. However, in cases where L-indices of the applicants are equal up to one decimal place, we strongly suggest the use of peer review, involving thorough examination of their publications, rather than differentiation of scientists based on the second decimal place, to avoid false precision and statistical bias. In case of young researchers that have only a few citations, it is also advisable to use peer review, as the limited data do not allow for the statistically robust calculation of the citation index.

The L-index can increase or decrease with time, as it depends on the age of publications. Thus, it favors the impact of recent publications and gives a much needed advantage to younger researchers. However, if a scientist has made such a significant discovery that its impact only increases with time, his L-index will stay high regardless of the age of the publication. Perfect examples of this are John von Neumann, Albert Einstein, Lev Landau, Alan Turing, Charles Darwin, Richard Feynman and Werner Heisenberg. Despite them ceasing to publish original work decades ago, their L-indices are still higher than those of the majority of current researchers ( Figure 2). It has to be noted that reprints of their seminal works or even full collections of works in modern journals contribute significantly to their L-indices because the age of the paper in the denominator is dramatically reduced whereas the original work is already very well known in the scientific community, so the fresh reprint immediately attracts lots of citations. More importantly, as Google Scholar (or the curator of the scientist’s profile) often merges the reprints of the same work from different years into one, it sometimes assigns the year of the latest reprint instead of the year of the original publication to the merged entity. This can dramatically affect the L-index, as all citations accumulated over decades are now divided by a very young age of the modern reprint. We had to exclude a few prominent scientists of the past from our analysis due to this issue. However, this is the problem of the database curation, not the L-index.

To make sure that 100 most cited articles capture the bulk of the L-index value, we utilized our dataset of reports for 45 prominent scientists ^19^, each containing top publications sorted by their contribution to the L-index which we called the score:


$$s_{i}=\frac{c_{i}}{a_{i}y_{i}}\;\text{(}4\text{)}$$

where *s _i_* – the score of *i*-th publication, *c _i_* – number of citations to *i*-th publication, *a _i_* – number of authors of *i*-th publication, *y _i_* – age in years of *i*-th publication.

**Figure 3.**
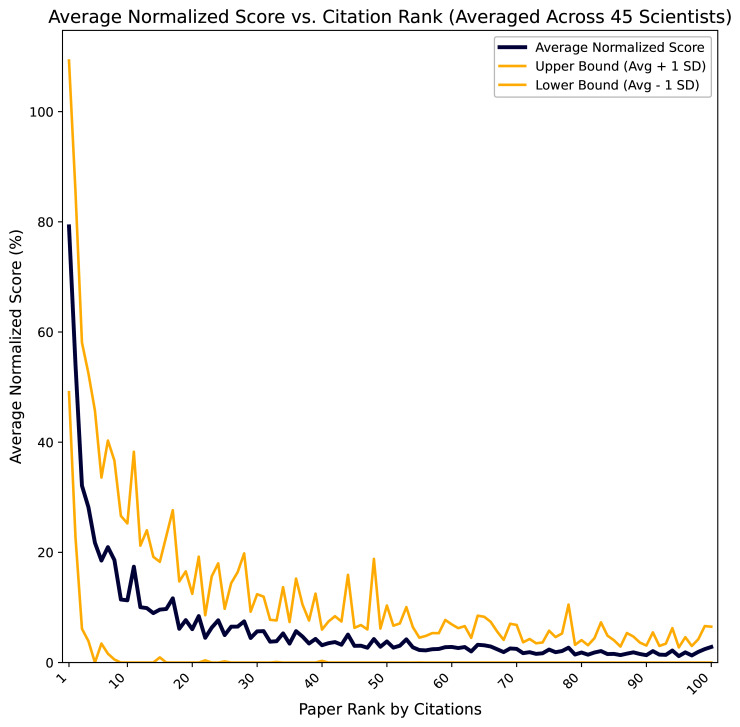
Average normalized score vs. citation rank. Scores and citations were calculated on the 8 ^th^ of May 2025 using the L-index Calculator ^18^ for 100 most cited publications from publicly available Google Scholar profiles of 45 prominent scientists in biomedicine, neuroscience, physics and computer science. The score of a publication is the number of citations divided by the number of authors and age in years. For each scientist separately, the scores for each paper were normalized relative to the highest scoring paper for that scientist. The papers were sorted by citations and the normalized scores averaged for all papers with the same citation rank. The reports for each scientist containing top publications sorted by scores are available as an external dataset ^19^.

For each scientist separately, we first normalized the scores for each paper relative to the highest scoring paper for that scientist (the first place in the report). Because each paper also has a citation value, we now sorted the papers by citations but kept the associated score values. We then averaged the normalized scores for all papers ranking 1 ^st^ by citations across all 45 scientists, for all papers ranking 2 ^nd^ etc. Finally, we plotted the averaged normalized scores vs paper rank by citations ( Figure 3).

It can be clearly seen that contribution to the L-index, which is essentially the logarithm of the sum of paper scores, drops rapidly with increasing citation rank, due to the power law distribution of citations ^13^. Thus, using 100 most cited papers gives plenty of headroom to capture almost all of the important signal. Even top 50 papers are sufficient for most cases. Nevertheless, scientists should be compared based on the same number of top publications used for calculating their L-indices.

The quantitative comparison of the L-index with other evaluation indices, such as the h-index and its derivatives, was purposefully avoided in this article, for the reason that those indices have been designed on different premises, such as to account for the number of publications. When evaluating the performance of a researcher, it should first be decided which parameter is considered adequate for the purpose – the number of publications, which does not tell anything about their quality, or the number of citations, which, however indirectly, indicates the impact that the publications have made. If the latter option is selected, the L-index can help to account for the effects of multiple co-authorship and publication age, and present the results in a simple and intuitive form.

## Data Availability

Analysis code available from: https://github.com/alekseybelikov/L-index Archived analysis code as at time of publication: Belikov A: L-index Calculator: A Python tool for academic citation analysis (v1.0) [Software]. *Zenodo*. 2025; https://doi.org/10.5281/zenodo.15356378 Analysis code license: GNU Affero General Public License v3.0 (AGPL-3.0) Analysis results available at: Belikov A: L-index reports for selected prominent scientists [Data set]. *Zenodo*. 2025; https://doi.org/10.5281/zenodo.15368429 • Analysis results license: Creative Commons Attribution 4.0 International (CC BY 4.0)
