# Supplementary material for: A citation-based, author- and age-normalized, logarithmic index for evaluation of individual researchers independently of publication counts
Source: F1000Res. 2025 Jun 2;4:884. Originally published 2015 Sep 22. [Version 2] doi: 10.12688/f1000research.7070.2 (PMC4654436; doi:10.12688/f1000research.7070.2)
Supplement: Peer review history

# Reviewer report: Reviewer response for version 1

**DOI:** 10.5256/f1000research.7610.r10836

**Published:** 2015-11-17

**Authors:** Danielle M. Colbert-Lewis

**Affiliations:** 1 Department of James E. Shepard Memorial Library, North Carolina Central University, Durham, NC, USA

This title is appropriate for the content of the article and the abstract represents a suitable summary for the work. The design, methods and analysis used in the article represented a thorough approach to the subject. The conclusions are suitable, balanced and justified on the basis of the results of the study. The data represented allows enough information to replicate the experiment for further research. The data is usable with the structure that has been provided.

Reviewer Expertise:

NA

I confirm that I have read this submission and believe that I have an appropriate level of expertise to confirm that it is of an acceptable scientific standard.

# Reviewer report: Reviewer response for version 1

**DOI:** 10.5256/f1000research.7610.r10421

**Published:** 2015-10-16

**Authors:** Youngim Jung

**Affiliations:** 1 Korea Institute of Science and Technology Information, Daejeon, South Korea

This paper proposes a novel index based on the number of citations, normalized by the number of authors and the date of publication, which is then scaled from 0.0 to 9.9 using the natural logarithm. The index proposed is intuitively designed in a simple way for readers to understand, with a fractional counting scheme adopted to normalize the number of citations, which appears reasonable. However, it will be necessary to adjust the index according to the relationship between the number of citations and the date of publication, since they are not precisely inversely proportional in every time window.

Finally and fundamentally, whether the number of authors and the date of publication are truly indicative of publication quality remains open to speculation.

Reviewer Expertise:

NA

I confirm that I have read this submission and believe that I have an appropriate level of expertise to confirm that it is of an acceptable scientific standard.
